# Supplementary material for: Achieving universal health coverage for people with stroke in South Africa: protocol for a scoping review
Source: BMJ Open. 2020 Oct 12;10(10):e041221. doi: 10.1136/bmjopen-2020-041221 (PMC7552861; doi:10.1136/bmjopen-2020-041221)
Supplement: Supplementary data [file bmjopen-2020-041221supp001.pdf]

Achieving Universal Health Coverage for people with stroke in South Africa: Protocol for a scoping review

Supplementary File:

Definitions

Universal health coverage **-** "Universal health coverage means that all people receive the health services they need, including public health services designed to promote better health (such as anti-tobacco information campaigns and taxes), prevent illness (such as vaccinations), and to provide treatment, rehabilitation and palliative care (such as end-of-life care) of sufficient quality to be effective, while at the same time ensuring that the use of these services does not expose the user to financial hardship"[1].

Health System - "A health system consists of all organizations, people and actions whose *primary intent* is to promote, restore or maintain health. This includes efforts to influence the determinants of health as well as more direct health-improving activities. A health system is, therefore, more than the pyramid of publicly owned facilities that deliver personal health services"[2].

Health system goals – "Health systems have multiple goals. The World health report 2000 defined overall health system outcomes or goals such as improving health and health equity, in ways that are responsive, financially fair, and make the best, or most efficient, use of available resources. There are also important intermediate goals: the route from inputs to health outcomes is through achieving greater access to and coverage for effective health interventions, without compromising efforts to ensure provider quality and safety"[2].

Health System building blocks – "To achieve their goals, all health systems have to carry out some basic functions, regardless of how they are organized: they have to provide services; develop health workers and other key resources; mobilize and allocate finances, and ensure health system leadership and governance (also known as stewardship, which is about oversight and guidance of the whole system). For the purpose of clearly articulating what WHO will do to help strengthen health systems, the functions identified in the World health report 2000 have been broken down into a set of six essential 'building blocks'. All are needed to improve outcomes [2].

Health system strengthening - Is defined as improving the six health system building blocks and managing their interactions in ways that achieve more equitable and sustained improvements across health services and health outcomes. It requires both technical and political knowledge and action[2].

Stroke care- Is defined by the World Stroke Organisation as **"...**the continuum of care starting at the onset of a stroke event through the hyperacute phase, acute inpatient care, stroke rehabilitation, prevention of recurrent stroke and concludes with community reintegration and long term recovery" [3].

Clinical guideline - Clinical guidelines are statements that include recommendations intended to optimise patient care that are informed by a systematic review of evidence and an assessment of the benefits and harms of alternative care options [4].

References:

1. World Health Organisation: Tracking universal health coverage: 2017 global monitoring report. World Health Organization and International Bank for Reconstruction and Development / The World Bank; 2017. Licence: CC BY-NC-SA 3.0 IGO.

2. World Health Organization: Everybody's business: strengthening health systems to improve health outcomes. WHO's Framework for Action. Geneva: World Health Organization; 2007. http://wwwwhoint/healthsystems/strategy/everybodys_businesspdf.

3. Lindsay MP, Norrving B, Furie KL, Donnan G, Langhorne P, Davis S. Global stroke guidelines and action plan: a road map for quality stroke care. Geneva: World Stroke Organization. 2016.

4. Institute of Medicine (US). Committee on standards for developing trustworthy clinical practice guidelines. Clinical practice guidelines we can trust. 2011:53-6.
