# Supplementary material for: Achieving universal health coverage for people with stroke in South Africa: protocol for a scoping review
Source: BMJ Open. 2020 Oct 12;10(10):e041221. doi: 10.1136/bmjopen-2020-041221 (PMC7552861; doi:10.1136/bmjopen-2020-041221)
Supplement: Author's manuscript [file bmjopen-2020-041221.draft_revisions.pdf]

BMJ Open is committed to open peer review. As part of this commitment we make the peer review history of every article we publish publicly available.

When an article is published we post the peer reviewers' comments and the authors' responses online. We also post the versions of the paper that were used during peer review. These are the versions that the peer review comments apply to.

The versions of the paper that follow are the versions that were submitted during the peer review process. They are not the versions of record or the final published versions. They should not be cited or distributed as the published version of this manuscript.

BMJ Open is an open access journal and the full, final, typeset and author-corrected version of record of the manuscript is available on our site with no access controls, subscription charges or pay-per-view fees (http://bmjopen.bmj.com).

If you have any questions on BMJ Open's open peer review process please email info.bmjopen@bmj.com

BMJ Open

Achieving Universal Health Coverage for people with stroke in South Africa:Protocol for a scoping review

Journal:	*BMJ Open*
Manuscript ID	bmjopen-2020-041221
Article Type:	Protocol
Date Submitted by the Author:	03-Jun-2020

Complete List of Authors:	Sjan-Mari, van Niekerk; Stellenbosch University, Division of Physiotherapy, Department of Health and Rehabilitation Sciences<br>Gakeemah, Inglis-Jassiem; Stellenbosch University, Division of Physiotherapy, Department of Health and Rehabilitation Sciences<br>Sureshkumar, K; Public Health Foundation of India, SACDIR; London School of Hygiene and Tropical Medicine, International Center for Evidence in Disability<br>Fernandes, Silke; London School of Hygiene and Tropical Medicine,<br>Webster, Jayne; London School of Hygiene and Tropical Medicine,<br>English, Rene; Stellenbosch University Faculty of Medicine and Health Sciences, Global Health<br>Smythe, Tracey; London School of Hygiene and Tropical Medicine, Clinical Research<br>Louw, QA; Faculty of Health Sciences, Stellenbosch University,

Keywords:	Stroke < NEUROLOGY, PUBLIC HEALTH, HEALTH ECONOMICS, Health policy < HEALTH SERVICES ADMINISTRATION & MANAGEMENT

SCHOLARONE™
Manuscripts

I, the Submitting Author has the right to grant and does grant on behalf of all authors of the Work (as defined in the below author licence), an exclusive licence and/or a non-exclusive licence for contributions from authors who are: i) UK Crown employees; ii) where BMJ has agreed a CC-BY licence shall apply, and/or iii) in accordance with the terms applicable for US Federal Government officers or employees acting as part of their official duties; on a worldwide, perpetual, irrevocable, royalty-free basis to BMJ Publishing Group Ltd ("BMJ") its licensees and where the relevant Journal is co-owned by BMJ to the co-owners of the Journal, to publish the Work in this journal and any other BMJ products and to exploit all rights, as set out in our licence.

The Submitting Author accepts and understands that any supply made under these terms is made by BMJ to the Submitting Author unless you are acting as an employee on behalf of your employer or a postgraduate student of an affiliated institution which is paying any applicable article publishing charge ("APC") for Open Access articles. Where the Submitting Author wishes to make the Work available on an Open Access basis (and intends to pay the relevant APC), the terms of reuse of such Open Access shall be governed by a Creative Commons licence – details of these licences and which Creative Commons licence will apply to this Work are set out in our licence referred to above.

Other than as permitted in any relevant BMJ Author's Self Archiving Policies, I confirm this Work has not been accepted for publication elsewhere, is not being considered for publication elsewhere and does not duplicate material already published. I confirm all authors consent to publication of this Work and authorise the granting of this licence.

For peer review only

Achieving Universal Health Coverage for people with stroke in South Africa: Protocol for a scoping review

Authors:

*Sjan-Mari van Niekerk[1], Gakeemah Inglis-Jassiem[1], Sureshkumar Kamalakannan[2], Silke Fernandes[4], Jayne Webster[3], Rene English[5],Tracey Smythe[2], Quinette Louw[1]

Corresponding Author

Sjan-Mari van Niekerk

Stellenbosch University

Faculty of Medicine and Health Sciences

Division of Physiotherapy

PO Box 241, Cape Town 8000, South Africa

E-mail: smbrown@sun.ac.za

Author Affiliations:

[1] Division of Physiotherapy, Department of Health and Rehabilitation Sciences, Faculty of Medicine and Health Sciences, Stellenbosch University , P. O. BOX 241, Cape Town, 8000, South Africa.

[2] International Centre for Evidence in Disability, London School of Hygiene & Tropical Medicine, Keppel st, WC1E7HT, U.K

[3] Department of Disease Control, London School of Hygiene & Tropical Medicine, Keppel st, WC1E7HT, U.K

[4] Department of Global Health and Development, London School of Hygiene & Tropical Medicine, 15-17 Tavistock Place, WC1H 9SH, UK

[5] Division of Health Systems and Public Health, Department of Global Health, Faculty of Medicine and Health Sciences, Stellenbosch University, Stellenbosch, South Africa

Total word Count – 3723

Key words – Stroke care; UHC; Health system; South Africa

For peer review only

Abstract

Introduction:

Stroke is the second most common cause of death after HIV/AIDS and a significant health burden in South Africa. The extent to which Universal Health Coverage is achieved for people with stroke in South Africa is unknown. Therefore a scoping review to explore the opportunities and challenges within the South African health system to facilitate the achievement of UHC for people with stroke is warranted.

Methods and analysis:

The scoping review will follow the approach recommended by Levac, Colquhoun and O'Brien, which includes five-steps: 1) identifying the research question, 2) identifying relevant studies, 3) selecting the studies, 4) charting the data, and 5) collating, summarising and reporting the results. Health Systems Dynamics Framework and WHO Framework on integrated people-centred health services will be used to map, synthesise and analyse data thematically.

Ethics and dissemination:

Ethical approval is not required for this scoping review, as it will only include published and publicly available data. The findings of this review will be published in an open-access, peer-reviewed journal and we will develop an accessible summary of the results for website posting and stakeholder meetings. Data generated from this review will be made available upon reasonable request.

Strengths and limitations of this study:

- The study will apply a scoping review methodology to evaluate the extent to which Universal Health Care (UHC) for people with stroke is achieved in South Africa
- In-depth primary analysis using the Health Systems Dynamics Framework and WHO Framework on integrated people-centred health services will map the data
- The study will determine opportunities and challenges within the South African health system to facilitate the achievement of UHC for people with stroke.
- The review is limited to papers published in English between 2005 and 2020

For peer review only

Definitions

Universal health coverage **-** “Universal health coverage means that all people receive the health services they need, including public health services designed to promote better health (such as anti-tobacco information campaigns and taxes), prevent illness (such as vaccinations), and to provide treatment, rehabilitation and palliative care (such as end-of-life care) of sufficient quality to be effective, while at the same time ensuring that the use of these services does not expose the user to financial hardship”[1].

Health System - “A health system consists of all organizations, people and actions whose *primary intent* is to promote, restore or maintain health. This includes efforts to influence the determinants of health as well as more direct health-improving activities. A health system is, therefore, more than the pyramid of publicly owned facilities that deliver personal health services"[2].

Health system goals – “Health systems have multiple goals. The World health report 2000 defined overall health system outcomes or goals such as improving health and health equity, in ways that are responsive, financially fair, and make the best, or most efficient, use of available resources. There are also important intermediate goals: the route from inputs to health outcomes is through achieving greater access to and coverage for effective health interventions, without compromising efforts to ensure provider quality and safety"[2].

Health System building blocks – “To achieve their goals, all health systems have to carry out some basic functions, regardless of how they are organized: they have to provide services; develop health workers and other key resources; mobilize and allocate finances, and ensure health system leadership and governance (also known as stewardship, which is about oversight and guidance of the whole system). For the purpose of clearly articulating what WHO will do to help strengthen health systems, the functions identified in the World health report 2000 have been broken down into a set of six essential ‘building blocks’. All are needed to improve outcomes [2].

Health system strengthening - Is defined as improving the six health system building blocks and managing their interactions in ways that achieve more equitable and sustained improvements across health services and health outcomes. It requires both technical and political knowledge and action[2].

For peer review only

Stroke care- Is defined by the World Stroke Organisation as **"...**the continuum of care starting at the onset of a stroke event through the hyperacute phase, acute inpatient care, stroke rehabilitation, prevention of recurrent stroke and concludes with community reintegration and long term recovery" [3].

Clinical guideline - Clinical guidelines are statements that include recommendations intended to optimise patient care that are informed by a systematic review of evidence and an assessment of the benefits and harms of alternative care options [4].

For peer review only

Introduction

Stroke is the second most common cause of death after HIV/AIDS and a significant health burden in South Africa (SA) [5-8]; an estimated 75000 strokes occur each year in SA, with a projected burden of disease of 564000 DALYs [9]. The burden of stroke in rural areas of South Africa is also on the increase with an estimated 33500 strokes occurring in 2011 in these areas alone [10]. However, the number of people living with stroke in SA is likely underestimated as no national stroke database or registry is in place, and these estimates are calculated from limited studies undertaken in a few parts of the country.

Stroke is the leading cause of disability in adults in SA, placing additional strain on social and healthcare services nationally [11]. Increased (i) prevalence of cardiovascular risk factors (such as hypertension, obesity, diabetes mellitus), (ii) unchecked industrialisation and (iii) urbanisation, contribute to this epidemiological transition of stroke in many low and middle-income countries [12], including SA [5]. Cardiovascular diseases, including stroke, was previously set to surpass infectious diseases as the major cause of morbidity and mortality in sub-Saharan Africa [13] with the burden of these non-communicable diseases increasing in this region of Africa [14]. In the last decade, a growing body of evidence shows an association with non-communicable diseases and infectious diseases: for example, diabetes mellitus, chronic respiratory conditions and chronic kidney disease have been linked to increased tuberculosis (TB) morbidity and mortality and vice versa [15]. Non-communicable diseases may cause "…impaired immunity, metabolic imbalances and stress factors which favour the manifestation of TB" [15]. Furthermore, younger individuals in their most economically productive years are increasingly being affected in Africa, more often presenting with infectious comorbidities such as HIV/AIDS or TB in addition to stroke [16, 17], which further increases the burden on already strained health systems. With the recent COVID-19 global pandemic, it had become evident that individuals with underlying chronic conditions experience more complications and require more complex treatments when infected with the novel virus [18].

Care for people with stroke

The delivery of effective and efficient stroke care is crucial to enhance the physical, cognitive and emotional well-being of individuals post-stroke, to improve their functional independence and quality of life, and ultimately to enable reintegration to their communities [19, 20]. For the purposes of this review, we define stroke care as **"…**the continuum of care starting at the onset of a stroke event through the hyperacute phase, acute inpatient care, stroke rehabilitation, prevention of recurrent stroke and concludes with community reintegration and long term recovery" [3].

For peer review only

Internationally, delivering effective stroke care has changed over the years as an understanding of stroke has increased, moving from individual care to a more comprehensive health system approach [21].

The majority of people with stroke in SA may have no or limited access to stroke care and rehabilitation [7, 22]. Stroke rehabilitation is limited in the first instance by insufficient rehabilitation facilities in the public sector, as well as inadequate intensity of inpatient rehabilitation, and lack of outpatient or community-based rehabilitation [23]. Reduced access to outpatient community-based stroke rehabilitation services was shown in a predominantly urban province of SA, where people with stroke only received 1–4 hours of physiotherapy sessions with a median number of 1.8 hours over a 6-month period [24]. In SA, stroke care occurs across a range of settings, from tertiary hospitals to remote community primary healthcare facilities; care for people with stroke can be provided individually or in a group setting, at home, in a community environment or a specialist centre [5]. Whilst public health policy in SA ascribes to primary health care and a decentralised approach, many stroke care and rehabilitation services remain centralised at district and specialist rehabilitation hospitals [25], which reinforces the inequality experienced by rural communities in terms of healthcare access [5]. Although home-based care services are available in certain communities, poor referral to and articulation with these services and other healthcare levels, often result in poor service delivery [26]. Individuals who experience mobility limitations post-stroke subsequently experience restricted access to services, as do those with minimal access to transport. Free primary level rehabilitation services are available in some communities, however these services may not be comprehensive or efficient due to shortages of staff skilled in stroke care or incomplete multidisciplinary teams [25]. People with stroke may be discharged home without receiving rehabilitation interventions, and families experience both catastrophic health expenditure and financial loss due to additional responsibilities of caregiving, often leading to an inability to maintain gainful employment [7,27]. As in other low- and middle-income countries, unmet stroke care and rehabilitation needs are further entrenched due to limited financial and infrastructural/architectural resources [23,27]. In addition, in SA there are no dedicated support systems for caregivers and people with stroke, which is compounded by a population that experience low levels of health literacy [24, 28, 29]. Health seeking behaviours may be further affected by the influence of religion and cultural beliefs on patients' views regarding the causes and management of cardiovascular conditions such as stroke, which highlights the need for culturally sensitive stroke care and patient-centric health systems [30].

For peer review only

Status of stroke care in South Africa

The socio-political context of SA has had significant effects on national healthcare policies and services, largely due to disparities in social and financial capital based on discriminatory racial and gender divides [8]. Although classified as a middle-income country, SA has high levels of poverty and unemployment, with unemployment rates ranging between 29% (nationally) and 50% (amongst younger people) and relative poverty worsening over time (Gini coefficient increased from 0.6 in 1995 to almost 0.7 in 2009) [31]. These social determinants of health influence the high levels of mortality and morbidity brought about by infectious diseases such as HIV and TB, as well as non-communicable diseases such as diabetes and hypertension [8,32]. Since the first democratic election in 1994, both government and society strive for equality and adopted a human-rights based approach to healthcare reform. Despite well-founded rights-based policy development with practical application, many areas still struggle to achieve equality [32]. The SA government has committed to the World Health Organisation (WHO) vision of achieving equitable, evidence-based rehabilitation for all by 2030 [1], however it remains "unclear how many South Africans access and receive rehabilitation services after sustaining a stroke, what rehabilitation is provided to them, how effective this rehabilitation is, and what the implications are, of receiving inadequate, or even no, rehabilitation" [29].

Universal health coverage for people with stroke in South Africa

The proposed National Health Insurance (NHI) Bill, currently being considered by the National Assembly of SA [33] focusses on primary health care as the foundation for Universal health coverage (UHC), however the Bill may not provide coverage for most age-related health conditions such as mobility impairments, and visual or auditory impairments [34]. UHC occurs where everyone receives essential services, according to their need and without financial hardship [35]. Essential services are defined as promotion, prevention, treatment, rehabilitation (including assistive technology) and palliative care. Key features of UHC include its human rights-focused underpinning and an integrated approach to health service delivery. It also importantly recognizes the role that health system functioning plays in the realization of UHC [36]. In recent years, SA has spearheaded radical healthcare policy reform to facilitate patient-centred and accessible service delivery as outlined in the Healthcare 2030 plan of the National Department of Health [29]. This has mainly been actioned via a decentralised district level healthcare service delivery model and the development of a NHI Scheme to curtail financial hardship experienced by users [37]. Moving towards UHC is a priority in SA and requires a strong and responsive health care delivery system to anchor effective and efficient service delivery. There is a crucial need for "good leadership, stewardship, and management of

For peer review only

health and related services to achieving health for all people" [8]. Louw et al. (2019) further affirm that "improving capacity across African healthcare settings is essential to ensure best practice health programmes to increase awareness of stroke and its causes, its early identification and acute management, and its rehabilitation" [38].

In general, stroke care services in Africa are not adequately supported by governments, with limited support for either the development or implementation of national stroke policy frameworks, or limited provision and endorsement of evidence-based clinical practice guidelines [39]. Where policy frameworks or practice guidelines are available, the implementation processes may not be well supported by states [40], which further limits best practice care for people with stroke [39]. In addition, uniquely African clinical practise guidelines for stroke care have not been well reported [41,42]. Practice guidelines that are appropriately adapted for local context are important, and consequently, the South African-contextualised Stroke Rehabilitation Guideline [25],developed in collaboration with key stakeholders, including the South African Department of Health, were developed between 2017-2018. This guideline marked a strategic and collaborative increased focus on the importance and quality of stroke rehabilitation in the SA healthcare context. However, the update of such guidelines in at all levels of care remain a challenge.

Accessible, responsive and quality stroke care services within a strengthened local health system will contribute to UHC for people with stroke and their caregivers in SA. The extent to which UHC is achieved for people with stroke in SA is unknown and relatively little is known about the opportunities and challenges within the local health system to achieve UHC.

Aim of this scoping review

The aim of this review is to explore the opportunities and challenges within the South African health system to facilitate the achievement of UHC for people with stroke. A scoping review method was chosen to address the broad nature of our question as well as identify knowledge gaps to inform further research. For the purpose of this scoping review, we will specifically explore the components and characteristics of the stroke-related health system as well as policies that potentially facilitate or hinder stroke care. We will draw on two frameworks (Health Systems Dynamics Framework) [43] and WHO Framework on integrated people-centred health services [44] to map the data from different sources in a thematic content analysis. This information is required to adequately plan for the healthcare needs of people with stroke in the local SA context.

For peer review only

Methods:

We will conduct this scoping review according to the approach recommended by Levac, Colquhoun and O'Brien (2010) [45], which includes five-steps: 1) identifying the research question, 2) identifying relevant studies, 3) selecting the studies, 4) charting the data, and 5) collating, summarising and reporting the results. The review will be reported according to the Preferred Reporting Items for Systematic Reviews and Meta-Analyses extension for Scoping Reviews (PRISMA-ScR) guidelines [46].

Analytical framework

Our review will be guided by an analytical framework adapted from the Health Systems Dynamics Framework (HSDF) [42] and WHO Framework on integrated people-centred health services (IPCHS) [43] to map and synthesise data from different sources in a thematic content analysis. The HSDF incorporates the WHO health system building blocks (i) service delivery; (ii) health workforce; (iii) information; (iv) medical products, vaccines and technologies; (v) financing; and (vi) leadership and governance, and highlights how values and principles drive the behaviour of people when making choices and engaging with processes of any health system. This HSDF framework offers an integrated view of a health system in that it acknowledges the social, economic, political context and determinants of health. The WHO IPCHS aims to support countries to achieve UHC by facilitating access to health services that are provided in a way that is coordinated around people's needs, respects their preferences, and are safe, effective, timely, affordable, and of acceptable quality. Our analytical framework will include all of the HSDF components and two components from the IPCHS: 1) Re-orientation of care and 2) Enabling environment, which are appropriate to the SA context and population (see Figure 1).

Step 1: Defining the review objectives

In line with the purpose of scoping reviews, our approach is broad, with emphasis on studies that investigate any aspect of the health care system in regard to stroke care in South Africa.

The review objectives are to:

1. describe the health system related factors such as governance, resources, community engagement, and service delivery that support and guide stroke care in South Africa
2. describe the health system-related factors such as governance, resources, community engagement that limit the achievement of universal stroke care in South Africa
3. synthesise the findings to gain insights into the driving factors that will fundamentally bring required change to achieve universal stroke care in South Africa

For peer review only

Step 2: Identifying relevant studies

Search Strategy

A comprehensive search, with the most appropriate search terms per database, will be conducted for each of the following databases: PubMed, EBSCOhost (including Medline AND CINAHL), Scopus, Global Health and African-wide databases (including EBSCOHost Africa Wide, African Journals Sabinet and African Journals Online (AJOL)). The databases were chosen to ensure that all relevant literature was identified. An initial, limited search selection of relevant databases will take place, followed by an analysis of text words in the title and abstract, as well as the index terms used to describe the article. To ensure that all relevant studies are included, additional, peer-reviewed literature will be added by hand searching the reference lists of the articles that were initially included to ensure that articles have not been missed.

Grey literature will be identified by using the WHO's OpenGrey and OpenDOAR Library, as well as sources such as Open Access Theses and Dissertations, Sabinet: Current and Completed Research, ProQuest Dissertations and Theses, Database of African Theses and Dissertations and the Networked Digital Library of Theses and Dissertations to include research conducted on stroke care in South Africa. The websites of relevant government and service provider agencies will be searched to identify relevant South African stroke care policy documents and/or practice guidance documents. In addition, field experts will be contacted to identify additional relevant evidence regarding stroke care in SA. Saturation will be the point at which no new evidence is found to be included in the scoping review.

A Boolean search string has been developed through the systematic process of reviewing Medical Subject Heading terms on Medline. A key term search strategy will be employed using a variety of combinations of terms for 'stroke', AND 'health system' OR 'Universal Health Coverage' OR 'clinical practice guideline' AND 'South Africa'. We will also conduct specific key term searches using each of the seven different components from the analytic framework (figure 1), combined with 'stroke' and 'South Africa'. We have conducted a pilot search in Google Scholar on the 9th of May 2020, to demonstrate the feasibility of answering our research question using a scoping review method. For the pilot search we have used the following combination of search terms 'stroke' AND 'South Africa' AND 'health system' OR 'Universal Health Coverage' OR 'clinical practice guideline' -cardiovascular -diabetes', which resulted in 2440 hits and a possible 100 publications were retrieved by title.

For peer review only

Eligibility criteria

This scoping review will include primary and secondary research studies, policy documents, reports and clinical practice guidelines describing any aspect of the health care system for people with stroke in SA. The review will include qualitative, quantitative and mixed methods studies of any study design. Records published or made available between 2005 and 2020 will be accepted for inclusion. A national reform of the SA health care system was called on in 2009, thus this timeframe would ensure that we include all possible studies that focused on the functioning of the SA health system since the time of reform until the present moment [47].

Records will be limited to those taking place in South Africa. Only records where the full text is available (in English or Afrikaans) will be included. Finally, evidence will be interrogated as to whether they addressed at least one of the components of the analytical framework.

Step 3: Evidence selection

Following database search, retrieved articles will be screened in three stages. First, one reviewer will independently screen the titles and abstracts of identified studies to exclude publications that do not meet the inclusion criteria. A second reviewer will check the results for accuracy. The data will be managed with Endnote X8. The full-text article will be retrieved for review if the citation is deemed eligible by at least one reviewer. Two of these reviewers will independently assess each article against the inclusion criteria. Any discrepancies between the reviewers will be resolved by discussion, and a third reviewer will be consulted if necessary. A PRISMA flow diagram will be completed to summarise the study selection process.

Step 4: Data charting

Data extraction will be undertaken independently by three reviewers (SvN, GIJ, SK). Each reviewer will be allocated specific data item(s) to extract, a second reviewer will check the extracted data for accuracy. Before commencing data extraction, the reviewers will first discuss the information to be extracted to ensure clarity of the data extraction process. Any discrepancies between the reviewers will be resolved by discussion, and a third reviewer will be consulted if necessary. A custom-designed form will be developed in Excel for data charting. Two independent reviewers will pilot the data charting form using a random sample of five included studies for consistency and required amendments will be agreed by consensus. We will modify the data extraction form as required based on feedback from the two reviewers, and the form amended at each stage where necessary.

For peer review only

We plan to contact study authors in the case of unclear information and will make up to three attempts by email.

Data items

The following general information will be extracted and tabulated from the included articles: author name(s), publishing journal, year of publication, region in South Africa, study population, the study aims/objectives/question, the setting, study-design, and findings. In addition, the following data will be extracted according to the components of HSDF and the WHO's IPCH framework components. To ensure consistency of the data extraction process, we will consider the seven components using the descriptions in Table 1.

Data synthesis and analysis

The analytical framework will guide the data synthesis and thematic analysis. We anticipate that the dataset will include different study designs, and therefore both descriptive statistics and narrative synthesis will be used. We will establish the strengths and opportunities that should be optimised in order to improve UHC of stroke services in South Africa as well as the weaknesses and threats which need to be minimised or eliminated.

Availability of data and materials: All data generated or analysed during this study will be included in the published scoping review article.

Conclusion

The aim of this review is to determine the successes and gaps of the current healthcare system to facilitate UHC for people with stroke in SA. The results of this review will be used to inform policymakers and healthcare professionals about healthcare systems challenges and opportunities as well as identify the potential future research areas that need to be considered in order to facilitate the focus areas for the successful implementation of NHI to effectively move towards UHC for people with stroke in South Africa.

Patient and Public Involvement:

No patients and public were not involved for the purpose of this scoping review.

For peer review only

Ethics and dissemination

Ethical approval is not required for this scoping review, as it will only include published and publicly available data. The findings of this review will be published in an open-access, peer-reviewed journal and we will develop an accessible summary of the results for website posting and stakeholder meetings. Data generated from this review will be made available upon reasonable request.

References

1. World Health Organisation: Tracking universal health coverage: 2017 global monitoring report. World Health Organization and International Bank for Reconstruction and Development / The World Bank; 2017. Licence: CC BY-NC-SA 3.0 IGO.

2. World Health Organization: Everybody's business: strengthening health systems to improve health outcomes. WHO's Framework for Action. Geneva: World Health Organization; 2007. http://wwwwhoint/healthsystems/strategy/everybodys_businesspdf.

3. Lindsay MP, Norrving B, Furie KL, Donnan G, Langhorne P, Davis S. Global stroke guidelines and action plan: a road map for quality stroke care. Geneva: World Stroke Organization. 2016.

4. Institute of Medicine (US). Committee on standards for developing trustworthy clinical practice guidelines. Clinical practice guidelines we can trust. 2011:53-6.

5. Scheffler E, Mash R. Surviving a stroke in South Africa: outcomes of home-based care in a low-resource rural setting. Topics in stroke rehabilitation. 2019 Aug 18;26(6):423-34.

6. Pillay-van Wyk V, Msemburi W, Laubscher R, Dorrington RE, Groenewald P, Glass T, Nojilana B, Joubert JD, Matzopoulos R, Prinsloo M, Nannan N. Mortality trends and differentials in South Africa from 1997 to 2012: second National Burden of Disease Study. Lancet Glob Health. 2016 Sep 1;4(9):e642-53.

7. Maredza M, Bertram MY, Gómez-Olivé XF, Tollman SM. Burden of stroke attributable to selected lifestyle risk factors in rural South Africa. BMC Public Health. 2016 Dec 1;16(1):143.

For peer review only

8. Coovadia H, Jewkes R, Barron P, Sanders D, McIntyre D. The health and health system of South Africa: historical roots of current public health challenges. The Lancet. 2009 Sep 5;374(9692):817-34.

9. Bertram MY, Katzenellenbogen J, Vos T, Bradshaw D, Hofman KJ. The disability adjusted life years due to stroke in South Africa in 2008. Int J Stroke. 2013 Oct;8(SA100):76-80.

10. Maredza M, Bertram MY, Tollman SM. Disease burden of stroke in rural South Africa: an estimate of incidence, mortality and disability adjusted life years. BMC Neurol 2015 Dec;15(1):54.

11. Salt is killing South Africans. The Heart and Stroke Foundation. 2015. https://www.health24.com/Diet-and-nutrition/News/Salt-is-killing-South-Africans-20130312.

12. Kabudula CW, Houle B, Collinson MA, Kahn K, Gómez-Olivé FX, Clark SJ, Tollman S. Progression of the epidemiological transition in a rural South African setting: findings from population surveillance in Agincourt, 1993–2013. BMC Public Health. 2017 Dec;17(1):424.

13. Yusuf S, Reddy S, Ôunpuu S, Anand S. Global burden of cardiovascular diseases: part I: general considerations, the epidemiologic transition, risk factors, and impact of urbanization. Circulation. 2001 Nov 27;104(22):2746-53.

14. Owolabi MO, Arulogun O, Melikam S, Adeoye AM, Akarolo-Anthony S, Akinyemi R, Arnett D, Tiwari H, Gebregziabher M, Jenkins C, Lackland D. The burden of stroke in Africa: a glance at the present and a glimpse into the future. Cardiovasc J Afr. 2015 Mar;26(2 H3Africa Suppl):S27.

15. Puchner KP, Rodriguez-Fernandez R, Oliver M, Solomos Z. Non-communicable diseases and tuberculosis: Anticipating the impending global storm. Glob Public Health. 2019 Sep 2;14(9):1372-81.

16. Zimba S, Ntanda PM, Lakhi S, Atadzhanov M. HIV infection, hypercoagulability and ischaemic stroke in adults at the University Teaching Hospital in Zambia: a case control study. BMC Infect Dis. 2017 Dec 1;17(1):354.

17. Heikinheimo T, Chimbayo D, Kumwenda JJ, Kampondeni S, Allain TJ. Stroke outcomes in Malawi, a country with high prevalence of HIV: a prospective follow-up study. PloS one. 2012;7(3).

For peer review only

18. Wu Z, McGoogan JM. Characteristics of and important lessons from the coronavirus disease 2019 (COVID-19) outbreak in China: summary of a report of 72 314 cases from the Chinese Center for Disease Control and Prevention. Jama. 2020 Apr 7;323(13):1239-42.

19. Kitzman P, Hudson K, Sylvia V, Feltner F, Lovins J. Care coordination for community transitions for individuals post-stroke returning to low-resource rural communities. Journal of community health. 2017 Jun 1;42(3):565-72.

20. Langhorne P, Bernhardt J, Kwakkel G. Stroke rehabilitation. The Lancet. 2011 May 14;377(9778):1693-702.

21. Miller KK, Lin SH, Neville M. From hospital to home to participation: a position paper on transition planning poststroke. Arch Phys Med Rehabil. 2019 Jun 1;100(6):1162-75.

22. Ntsiea MV. Current stroke rehabilitation services and physiotherapy research in South Africa. S Afr J Physiother. 2019;75(1).

23. Bryer A, Connor MD, Haug P, Cheyip B, Staub H, Tipping B, Duim WB, Pinkney-Atkinson V. The South African guideline for the management of ischemic stroke and transient ischemic attack: recommendations for a resource-constrained health care setting. Int J Stroke. 2011 Aug;6(4):349-54.

24. Rhoda A. *The rehabilitation of stroke patients at community health centres in the Western Cape* (Doctoral dissertation, University of the Western Cape).

25. South African Contextualised Stroke Rehabilitation Guideline (SA-CSRG). 2019. http://www.sun.ac.za/english/faculty/healthsciences/health-rehabilitation-sciences/Documents/Completed%20stroke%20guidelines_May2019.pdf

26. Mabunda SA, London L, Pienaar D. An evaluation of the role of an intermediate care facility in the continuum of care in Western Cape, South Africa. Int J Health Policy Manag. 2018 Feb;7(2):167.

27. Pandian JD, William AG, Kate MP, Norrving B, Mensah GA, Davis S, Roth GA, Thrift AG, Kengne AP, Kissela BM, Yu C. Strategies to improve stroke care services in low-and middle-income countries: a systematic review. Neuroepidemiology. 2017;49(1-2):45-61.

For peer review only

28. Visagie S, Swartz L. Rural South Africans' rehabilitation experiences: case studies from the Northern Cape Province. S Afr J Physiother. 2016;72(1).

29. Western Cape Government. Healthcare 2030: The Road to Wellness. Cape Town: Western Cape Department of Health; 2014.

30. Osokpo O, Riegel B. Cultural factors influencing self-care by persons with cardiovascular disease: An integrative review. Int J Nurs Stud. 2019 Jul 9:103383.

31. Benatar S, Gill S. Universal access to healthcare: the case of South Africa in the comparative global context of the late anthropocene era. International Journal of Health Policy and Management. 2020 Mar 1.

32. van den Heever AM. South Africa's universal health coverage reforms in the post-apartheid period. Health Policy. 2016 Dec 1;120(12):1420-8.

33. National Health Insurance Bill (https://www.gov.za/documents/national-health-insurance-bill-b-11-2019-6-aug-2019-0000)

34. McIntyre D, Garshong B, Mtei G, Meheus F, Thiede M, Akazili J, Ally M, Aikins M, Mulligan JA, Goudge J. Beyond fragmentation and towards universal coverage: insights from Ghana, South Africa and the United Republic of Tanzania. Bulletin of the World health Organization. 2008;86:871-6.

35. World Health Organisation: Health Systems Financing: The Path to Universal Coverage. World Health Report 2010. Geneva: WHO; 2010a.

36. Abiiro GA, De Allegri M. Universal health coverage from multiple perspectives: a synthesis of conceptual literature and global debates. BMC Int Health Hum Rights. 2015 Dec 1;15(1):17.

37. Fusheini A, Eyles J. Achieving universal health coverage in South Africa through a district health system approach: conflicting ideologies of health care provision. BMC Health Serv Res. 2016 Dec 1;16(1):558.

For peer review only

38. Louw Q, Twizeyemariya A, Grimmer K, Leibbrandt D. Estimating the costs and benefits of stroke rehabilitation in South Africa. J Eval Clin Pract. 2019 Sep 10.

39. Baatiema L, Otim M, Mnatzaganian G, Aikins AD, Coombes J, Somerset S. Towards best practice in acute stroke care in Ghana: a survey of hospital services. BMC Health Serv Res. 2017 Dec;17(1):108.

40. Cockburn L, Fanfon TN, Bramall A, Ngole EM, Kuwoh P, Anjonga E, Difang BM, Kiani S, Muso PS, Trivedi N, Sama J. Best practice guidelines for stroke in Cameroon: An innovative and participatory knowledge translation project. Afr J Disabil. 2014;3(1).

41. Okwen PM, Maweu I, Grimmer K, Margarita Dizon J. Evaluation of all African clinical practice guidelines for hypertension: Quality and opportunities for improvement. J Eval Clin Pract. 2019 Aug;25(4):565-74.

42. Grimmer K, Louw Q, Dizon JM, van Niekerk SM, Ernstzen D, Wiysonge C. Standardising evidence strength grading for recommendations from multiple clinical practice guidelines: a South African case study. Implementation Science. 2018 Dec 1;13(1):117.

43. Van Olmen J, Criel B, Bhojani U, Marchal B, Van Belle S, Chenge F, Hoeree T, Pirard M, Van Damme W, Kegels G. The health system dynamics framework: the introduction of an analytical model for health system analysis and its application to two case-studies. Health Culture and Society. 2012;2(1):1-21.

44. World Health Organization. Framework on integrated, people-centred health services. Report by the Secretariat. 69th World Health Assembly provisional agenda item. 2016 Apr 15;16:A69.

45. Levac D, Colquhoun H, O'Brien KK. Scoping studies: advancing the methodology. Implementation science. 2010 Dec 1;5(1):69.

46. Tricco AC, Lillie E, Zarin W, O'Brien KK, Colquhoun H, Levac D, Moher D, Peters MD, Horsley T, Weeks L, Hempel S. PRISMA extension for scoping reviews (PRISMA-ScR): checklist and explanation. Annals of internal medicine. 2018 Oct 2;169(7):467-73.

For peer review only

47. Mayosi BM, Lawn JE, Van Niekerk A, Bradshaw D, Karim SS, Coovadia HM, Lancet South Africa team. Health in South Africa: changes and challenges since 2009. The Lancet. 2012 Dec 8;380(9858):2029-43.

Contributions

SvN and GIJ drafted and revised the protocol with suggestions from TS, JW, SK, SF, RE and QAL who reviewed the protocol and provided feedback on the draft. SvN, SF and GIJ in consultation with the other authors constructed the search. All authors read and approved the final protocol.

Funding

This research was commissioned by the National Institute for Health Research (NIHR) NIHR Global Health Policy and Systems Research Development Award using UK aid from the UK Government. The views expressed in this publication are those of the author(s) and not necessarily those of the NIHR or the Department of Health and Social Care. Grant number NIHR130180.

Competing interests

None declared.

Figure Legends

Figure 1. Analytical framework (adapted from The Health Systems Dynamics Framework [43] and the WHO Framework on integrated people-centred health services components [44]

Table legends

Table 1: Application of the key characteristics of the analytical framework

For peer review only

Table 1: Application of the key characteristics of the analytical framework

FRAMEWORK COMPONENT	DESCRIPTION (data items)
**Governance/Regulation**	Healthcare policies at national or provincial levels; resource allocation policies; accountability; coordination and regulations.
**Resources**	Infrastructure (accessible; equipped and maintained); finance allocation and affordability; human resources (availability; distribution – occupation/specialisation, place of work, gender; graduates of health professionals); knowledge (clinical decision-making; clinical guidelines).
**Service Delivery**	Service delivery models; health services and service providers (private/public; for-profit or not–for-profit, formal or informal, professional or non-professional, allopathic or traditional, remunerated or voluntary).
**Context**	Socio-economic, technological, cultural, political and environmental environments.
**Re-orientation of care**	Health promotion and patient education. New technologies (eHealth; shared electronic medical records; telemedicine; m-health). Coordination of health services; continuous health care, multi-professional health teams; comprehensive health services and intersectoral coordination.
**Enabling environment**	Health workforce allocation/ shortages; distribution of health workforce; workforce training; clearly defined roles and fair wages.
**Population: Community engagement**	Engaging and empowering individuals, families, communities and informal carers. Reaching underserved and marginalised communities.<br>Common decision-making, self-efficacy of patients.

List of Abbreviations

UHC – Universal Health Coverage

SA – South Africa

WHO – World Health Organisation

IPCHS - Integrated people-centred health services

For peer review only

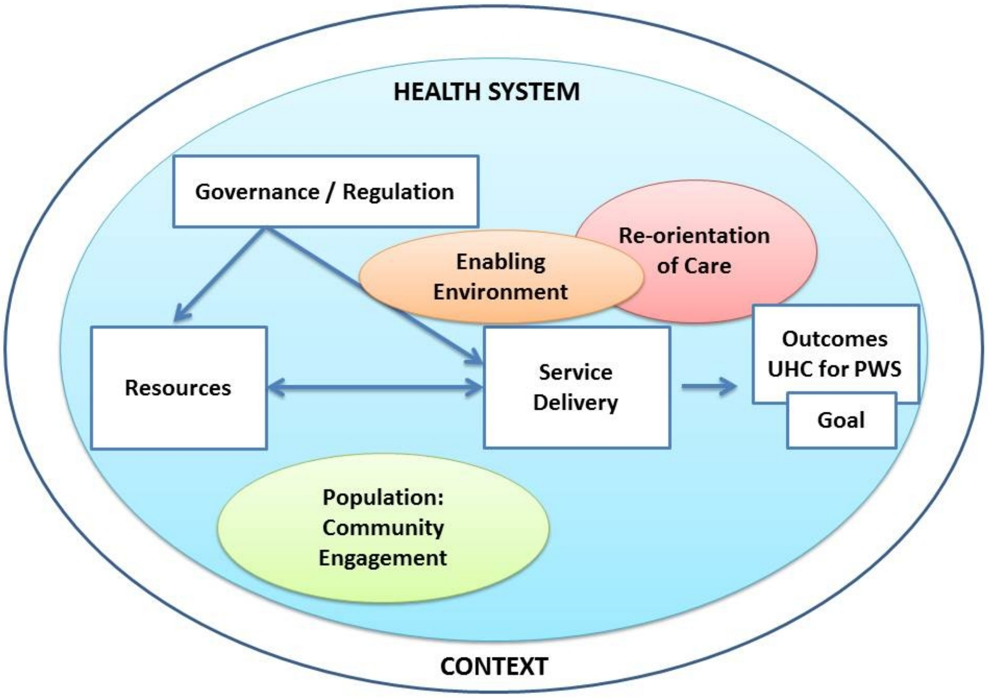

Figure-1: Analytical framework (adapted from The Health Systems Dynamics Framework and the WHO Framework on integrated people-centered health services components [44]

254x190mm (96 x 96 DPI)

BMJ Open

Achieving Universal Health Coverage for people with stroke in South Africa:Protocol for a scoping review

Journal:	*BMJ Open*
Manuscript ID	bmjopen-2020-041221.R1
Article Type:	Protocol
Date Submitted by the Author:	08-Sep-2020
Complete List of Authors:	Sjan-Mari, van Niekerk; Stellenbosch University, Division of Physiotherapy, Department of Health and Rehabilitation Sciences<br>Gakeemah, Inglis-Jassiem; Stellenbosch University, Division of Physiotherapy, Department of Health and Rehabilitation Sciences<br>Sureshkumar, K; Public Health Foundation of India, SACDIR; London School of Hygiene and Tropical Medicine, International Center for Evidence in Disability<br>Fernandes, Silke; London School of Hygiene and Tropical Medicine,<br>Webster, Jayne; London School of Hygiene and Tropical Medicine,<br>English, Rene; Stellenbosch University Faculty of Medicine and Health Sciences, Global Health<br>Smythe, Tracey; London School of Hygiene and Tropical Medicine, Clinical Research<br>Louw, QA; Faculty of Health Sciences, Stellenbosch University,
**Primary Subject Heading**:	Public health
Secondary Subject Heading:	Rehabilitation medicine, Neurology, Health services research, Health policy, Global health
Keywords:	PUBLIC HEALTH, HEALTH ECONOMICS, Stroke < NEUROLOGY, Health policy < HEALTH SERVICES ADMINISTRATION & MANAGEMENT

SCHOLARONE™
Manuscripts

I, the Submitting Author has the right to grant and does grant on behalf of all authors of the Work (as defined in the below author licence), an exclusive licence and/or a non-exclusive licence for contributions from authors who are: i) UK Crown employees; ii) where BMJ has agreed a CC-BY licence shall apply, and/or iii) in accordance with the terms applicable for US Federal Government officers or employees acting as part of their official duties; on a worldwide, perpetual, irrevocable, royalty-free basis to BMJ Publishing Group Ltd ("BMJ") its licensees and where the relevant Journal is co-owned by BMJ to the co-owners of the Journal, to publish the Work in this journal and any other BMJ products and to exploit all rights, as set out in our licence.

The Submitting Author accepts and understands that any supply made under these terms is made by BMJ to the Submitting Author unless you are acting as an employee on behalf of your employer or a postgraduate student of an affiliated institution which is paying any applicable article publishing charge ("APC") for Open Access articles. Where the Submitting Author wishes to make the Work available on an Open Access basis (and intends to pay the relevant APC), the terms of reuse of such Open Access shall be governed by a Creative Commons licence – details of these licences and which Creative Commons licence will apply to this Work are set out in our licence referred to above.

Other than as permitted in any relevant BMJ Author's Self Archiving Policies, I confirm this Work has not been accepted for publication elsewhere, is not being considered for publication elsewhere and does not duplicate material already published. I confirm all authors consent to publication of this Work and authorise the granting of this licence.

For peer review only

Achieving Universal Health Coverage for people with stroke in South Africa: Protocol for a scoping review

Authors:

*Sjan-Mari van Niekerk[1], Gakeemah Inglis-Jassiem[1], Sureshkumar Kamalakannan[2], Silke Fernandes[4], Jayne Webster[3], Rene English[5],Tracey Smythe[2], Louw QA[1]

Corresponding Author

Sjan-Mari van Niekerk

Stellenbosch University

Faculty of Medicine and Health Sciences

Division of Physiotherapy

PO Box 241, Cape Town 8000, South Africa

E-mail: smbrown@sun.ac.za

Author Affiliations:

[1] Division of Physiotherapy, Department of Health and Rehabilitation Sciences, Faculty of Medicine and Health Sciences, Stellenbosch University , P. O. BOX 241, Cape Town, 8000, South Africa.

[2] International Centre for Evidence in Disability, London School of Hygiene & Tropical Medicine, Keppel st, WC1E7HT, U.K

[3] Department of Disease Control, London School of Hygiene & Tropical Medicine, Keppel st, WC1E7HT, U.K

[4] Department of Global Health and Development, London School of Hygiene & Tropical Medicine, 15-17 Tavistock Place, WC1H 9SH, UK

[5] Division of Health Systems and Public Health, Department of Global Health, Faculty of Medicine and Health Sciences, Stellenbosch University, Stellenbosch, South Africa

Total word Count – 3509

Key words – Stroke care; UHC; Health system; South Africa

For peer review only

Abstract

Introduction:

Stroke is the second most common cause of death after HIV/AIDS and a significant health burden in South Africa. The extent to which Universal Health Coverage is achieved for people with stroke in South Africa is unknown. Therefore a scoping review to explore the opportunities and challenges within the South African health system to facilitate the achievement of Universal Health Coverage (UHC) for people with stroke is warranted.

Methods and analysis:

The scoping review will follow the approach recommended by Levac, Colquhoun and O'Brien, which includes five-steps: 1) identifying the research question, 2) identifying relevant studies, 3) selecting the studies, 4) charting the data, and 5) collating, summarising and reporting the results. Health Systems Dynamics Framework and WHO Framework on integrated people-centred health services will be used to map, synthesise and analyse data thematically.

Ethics and dissemination:

Ethical approval is not required for this scoping review, as it will only include published and publicly available data. The findings of this review will be published in an open-access, peer-reviewed journal and we will develop an accessible summary of the results for website posting and stakeholder meetings. Data generated from this review will be made available upon reasonable request.

Strengths and limitations of this study:

- A comprehensive scoping review methods is proposed to review the question
- Frameworks such as the Health Systems Dynamics and Integrated people-centred health services will be used.
- Global as well as National databases will be searched with comprehensive search strategies.
- The review will be limited to papers published between 2005 and 2020.
- The review will be limited to papers published in English and Afrikaans only.

For peer review only

Introduction

Stroke is the second most common cause of death after HIV/AIDS and a significant health burden in South Africa (SA) [1-4]; an estimated 75000 strokes occur each year in SA, with a projected burden of disease of 564000 Disability Adjusted Life Years (DALYs) [5]. The burden of stroke in rural areas of South Africa is also on the increase with an estimated 33500 strokes occurring in 2011 in these areas alone [6]. However, the number of people living with stroke in SA is likely underestimated as no national stroke database or registry is in place, and these estimates are calculated from limited studies undertaken in a few parts of the country.

Stroke is the leading cause of disability in adults in SA, placing additional strain on social and healthcare services nationally [7]. Increased (i) prevalence of cardiovascular risk factors (such as hypertension, obesity, diabetes mellitus), (ii) unchecked industrialisation and (iii) urbanisation, contribute to this epidemiological transition of stroke in many low and middle-income countries [8], including SA [1]. Cardiovascular diseases, including stroke, was previously set to surpass infectious diseases as the major cause of morbidity and mortality in sub-Saharan Africa [9] with the burden of these non-communicable diseases increasing in this region of Africa [10]. In the last decade, a growing body of evidence shows an association with non-communicable diseases and infectious diseases: for example, diabetes mellitus, chronic respiratory conditions and chronic kidney disease have been linked to increased tuberculosis (TB) morbidity and mortality and vice versa [11]. Non-communicable diseases may cause "…impaired immunity, metabolic imbalances and stress factors which favour the manifestation of TB" [11]. Furthermore, younger individuals in their most economically productive years are increasingly being affected in Africa, more often presenting with infectious comorbidities such as HIV/AIDS or TB in addition to stroke [12, 13], which further increases the burden on already strained health systems. With the recent COVID-19 global pandemic, it had become evident that individuals with underlying chronic conditions like stroke are more vulnerable to this infection and might have even more complex health and social care needs [14].

Care for people with stroke

The delivery of effective and efficient stroke care is crucial to enhance the physical, cognitive and emotional well-being of individuals post-stroke, to improve their functional independence and quality of life, and ultimately to enable reintegration to their communities [15, 16]. For the purposes of this review, we define stroke care as **"…**the continuum of care starting at the onset of a stroke

For peer review only

event through the hyperacute phase, acute inpatient care, stroke rehabilitation, prevention of recurrent stroke and concludes with community reintegration and long term recovery" [17]. Internationally, delivering effective stroke care has changed over the years as an understanding of stroke has increased, moving from individual care to a more comprehensive health system approach [18].

The majority of people with stroke in SA may have no or limited access to stroke care and rehabilitation [3, 19]. Stroke rehabilitation is limited in the first instance by insufficient rehabilitation facilities in the public sector, as well as inadequate intensity of inpatient rehabilitation, and lack of outpatient or community-based rehabilitation [20]. Reduced access to outpatient community-based stroke rehabilitation services was shown in a predominantly urban province of SA, where people with stroke only received 1–4 hours of physiotherapy sessions with a median number of 1.8 hours over a 6-month period [21]. In SA, stroke care occurs across a range of settings, from tertiary hospitals to remote community primary healthcare facilities; care for people with stroke can be provided individually or in a group setting, at home, in a community environment or a specialist centre [1]. Whilst public health policy in SA ascribes to primary health care and a decentralised approach, many stroke care and rehabilitation services remain centralised at district and specialist rehabilitation hospitals [22], which reinforces the inequality experienced by rural communities in terms of healthcare access [5]. Although home-based care services are available in certain communities, poor referral to and articulation with these services and other healthcare levels, often result in poor service delivery [23]. Individuals who experience mobility limitations post-stroke subsequently experience restricted access to services, as do those with minimal access to transport. Free primary level rehabilitation services are available in some communities, however these services may not be comprehensive or efficient due to shortages of staff skilled in stroke care or incomplete multidisciplinary teams [22]. People with stroke may be discharged home without receiving rehabilitation interventions, and families experience both catastrophic health expenditure and financial loss due to additional responsibilities of caregiving, often leading to an inability to maintain gainful employment [3,24]. As in other low- and middle-income countries, unmet stroke care and rehabilitation needs are further entrenched due to limited financial and infrastructural/architectural resources [20,24]. In addition, in SA there are no dedicated support systems for caregivers and people with stroke, which is compounded by a population that experience low levels of health literacy [21, 25, 26]. Health seeking behaviours may be further affected by the influence of religion and cultural beliefs on patients' views regarding the causes and management of cardiovascular

For peer review only

conditions such as stroke, which highlights the need for culturally sensitive stroke care and patient-centric health systems [27].

Status of stroke care in South Africa

The socio-political context of SA has had significant effects on national healthcare policies and services, largely due to disparities in social and financial capital based on discriminatory racial and gender divides [4]. Although classified as a middle-income country, SA has high levels of poverty and unemployment, with unemployment rates ranging between 29% (nationally) and 50% (amongst younger people) and relative poverty worsening over time (Gini coefficient increased from 0.6 in 1995 to almost 0.7 in 2009) [28]. These social determinants of health influence the high levels of mortality and morbidity brought about by infectious diseases such as HIV and TB, as well as non-communicable diseases such as diabetes and hypertension [4,29]. Since the first democratic election in 1994, both government and society strive for equality and adopted a human-rights based approach to healthcare reform. Despite well-founded rights-based policy development with practical application, many areas still struggle to achieve equality [29]. The SA government has committed to the World Health Organisation (WHO) vision of achieving equitable, evidence-based rehabilitation for all by 2030 [30], however it remains "unclear how many South Africans access and receive rehabilitation services after sustaining a stroke, what rehabilitation is provided to them, how effective this rehabilitation is, and what the implications are, of receiving inadequate, or even no, rehabilitation" [26].

Universal health coverage for people with stroke in South Africa

The proposed National Health Insurance (NHI) Bill, currently being considered by the National Assembly of SA [31] focusses on primary health care as the foundation for UHC , however the Bill may not provide coverage for most age-related health conditions such as mobility impairments, and visual or auditory impairments [32]. UHC occurs where everyone receives essential services, according to their need and without financial hardship [33]. Essential services are defined as promotion, prevention, treatment, rehabilitation (including assistive technology) and palliative care. Key features of UHC include its human rights-focused underpinning and an integrated approach to health service delivery. It also importantly recognizes the role that health system functioning plays in the realization of UHC [34]. In recent years, SA has spearheaded radical healthcare policy reform to facilitate patient-centred and accessible service delivery as outlined in the Healthcare 2030 plan of the National Department of Health [26]. This has mainly been actioned via a decentralised district level healthcare service delivery model and the development of a NHI Scheme to curtail financial

For peer review only

hardship experienced by users [35]. Moving towards UHC is a priority in SA and requires a strong and responsive health care delivery system to anchor effective and efficient service delivery. There is a crucial need for "good leadership, stewardship, and management of health and related services to achieving health for all people" [4]. Louw et al. (2019) further affirm that "improving capacity across African healthcare settings is essential to ensure best practice health programmes to increase awareness of stroke and its causes, its early identification and acute management, and its rehabilitation" [36].

In general, stroke care services in Africa are not adequately supported by governments, with limited support for either the development or implementation of national stroke policy frameworks, or limited provision and endorsement of evidence-based clinical practice guidelines [37]. Where policy frameworks or practice guidelines are available, the implementation processes may not be well supported by states [38], which further limits best practice care for people with stroke [37]. In addition, uniquely African clinical practise guidelines for stroke care have not been well reported [39, 40]. Practice guidelines that are appropriately adapted for local context are important, and consequently, the South African-contextualised Stroke Rehabilitation Guideline [22],developed in collaboration with key stakeholders, including the South African Department of Health, were developed between 2017-2018. This guideline marked a strategic and collaborative increased focus on the importance and quality of stroke rehabilitation in the SA healthcare context. However, the update of such guidelines in at all levels of care remain a challenge.

Accessible, responsive and quality stroke care services within a strengthened local health system will contribute to UHC for people with stroke and their caregivers in SA. The extent to which UHC is achieved for people with stroke in SA is unknown and relatively little is known about the opportunities and challenges within the local health system to achieve UHC.

Aim of this scoping review

The aim of this review is to explore the opportunities and challenges within the South African health system to facilitate the achievement of UHC for people with stroke. A scoping review method was chosen to address the broad nature of our question as well as identify knowledge gaps to inform further research. For the purpose of this scoping review, we will specifically explore the components and characteristics of the stroke-related health system as well as policies that potentially facilitate or hinder stroke care. We will draw on two frameworks (Health Systems Dynamics Framework) [41] and WHO Framework on integrated people-centred health services [42] to map the data from

For peer review only - http://bmjopen.bmj.com/site/about/guidelines.xhtml

different sources in a thematic content analysis. This information is required to adequately plan for the healthcare needs of people with stroke in the local SA context.

Methods:

We will conduct this scoping review according to the approach recommended by Levac, Colquhoun and O'Brien (2010) [43], which includes five-steps: 1) identifying the research question, 2) identifying relevant studies, 3) selecting the studies, 4) charting the data, and 5) collating, summarising and reporting the results. The review will be reported according to the Preferred Reporting Items for Systematic Reviews and Meta-Analyses extension for Scoping Reviews (PRISMA-ScR) guidelines using a modified PRISMA flow chart [44].

Analytical framework

Our review will be guided by an analytical framework adapted from the Health Systems Dynamics Framework (HSDF) [40] and WHO Framework on integrated people-centred health services (IPCHS) [41] to map and synthesise data from different sources in a thematic content analysis. The HSDF incorporates the WHO health system building blocks (i) service delivery; (ii) health workforce; (iii) information; (iv) medical products, vaccines and technologies; (v) financing; and (vi) leadership and governance, and highlights how values and principles drive the behaviour of people when making choices and engaging with processes of any health system. This HSDF framework offers an integrated view of a health system in that it acknowledges the social, economic, political context and determinants of health. The WHO IPCHS aims to support countries to achieve UHC by facilitating access to health services that are provided in a way that is coordinated around people's needs, respects their preferences, and are safe, effective, timely, affordable, and of acceptable quality. Our analytical framework will include all of the HSDF components and two components from the IPCHS: 1) Re-orientation of care and 2) Enabling environment, which are appropriate to the SA context and population (see Figure 1).

Step 1: Defining the review objectives

In line with the purpose of scoping reviews, our approach is broad, with emphasis on studies that investigate any aspect of the health care system in regard to stroke care in South Africa. The definitions of the concepts that will be used for this review is provided in supplementary file -1.

The review objectives are to:

1. describe the health system related factors such as governance, resources, community engagement, and service delivery that support and guide stroke care in South Africa

For peer review only

2. describe the health system-related factors such as governance, resources, community engagement that limit the achievement of universal stroke care in South Africa
3. synthesise the findings to gain insights into the driving factors that will fundamentally bring required change to achieve universal stroke care in South Africa

Step 2: Identifying relevant studies

Search Strategy

A comprehensive search, with the most appropriate search terms per database, will be conducted for each of the following databases: PubMed, EBSCOhost (including Medline AND CINAHL), Scopus, Global Health and African-wide databases (including EBSCOHost Africa Wide, African Journals Sabinet and African Journals Online (AJOL)). The databases were chosen to ensure that all relevant literature was identified. An initial, limited search selection of relevant databases will take place, followed by an analysis of text words in the title and abstract, as well as the index terms used to describe the article. To ensure that all relevant studies are included, additional, peer-reviewed literature will be added by hand searching the reference lists of the articles that were initially included to ensure that articles have not been missed. The search strategy developed for the purpose of the proposed review is provided in supplementary file – 2.

Grey literature will be identified by using the WHO's OpenGrey and OpenDOAR Library, as well as sources such as Open Access Theses and Dissertations, Sabinet: Current and Completed Research, ProQuest Dissertations and Theses, Database of African Theses and Dissertations and the Networked Digital Library of Theses and Dissertations to include research conducted on stroke care in South Africa. The websites of relevant government and service provider agencies will be searched to identify relevant South African stroke care policy documents and/or practice guidance documents. In addition, field experts will be contacted to identify additional relevant evidence regarding stroke care in SA. Saturation will be the point at which no new evidence is found to be included in the scoping review.

A Boolean search string has been developed through the systematic process of reviewing Medical Subject Heading terms on Medline. A key term search strategy will be employed using a variety of combinations of terms for 'stroke', AND 'health system' OR 'Universal Health Coverage' OR 'clinical practice guideline' AND 'South Africa'. We will also conduct specific key term searches using each of the seven different components from the analytic framework (figure 1), combined with 'stroke' and 'South Africa'. We have conducted a pilot search in Google Scholar on the 9th of May 2020, to demonstrate the feasibility of answering our research question using a scoping review method. For the pilot search we have used the following combination of search terms 'stroke' AND 'South Africa'

For peer review only

AND ‘health system’ OR ‘Universal Health Coverage’ OR ‘clinical practice guideline’ -cardiovascular -diabetes’, which resulted in 2440 hits and a possible 100 publications were retrieved by title.

Eligibility criteria

This scoping review will include primary and secondary research studies, policy documents, reports and clinical practice guidelines describing any aspect of the health care system for people with stroke in SA. The review will include qualitative, quantitative and mixed methods studies of any study design. Records published or made available between 2005 and 2020 will be accepted for inclusion. A national reform of the SA health care system was called on in 2009, thus this timeframe would ensure that we include all possible studies that focused on the functioning of the SA health system since the time of reform until the present moment [45].

Records will be limited to those taking place in South Africa. Only records where the full text is available (in English or Afrikaans) will be included. This is especially because the research team possess these language skills and its contextually relevant. Finally, evidence will be interrogated as to whether they addressed at least one of the components of the analytical framework.

Step 3: Evidence selection

Following database search, retrieved articles will be screened in three stages. First, one reviewer will independently screen the titles and abstracts of identified studies to exclude publications that do not meet the inclusion criteria. A second reviewer will check the results for accuracy. The data will be managed with Endnote X8. The full-text article will be retrieved for review if the citation is deemed eligible by at least one reviewer. Two of these reviewers will independently assess each article against the inclusion criteria. Any discrepancies between the reviewers will be resolved by discussion, and a third reviewer will be consulted if necessary. A Modified PRISMA that incorporates PRISMA-ScR reporting framework will be completed to summarise the study selection process.

Step 4: Data charting

Data extraction will be undertaken independently by three reviewers (SvN, GIJ, SK). Each reviewer will be allocated specific data item(s) to extract, a second reviewer will check the extracted data for accuracy. Before commencing data extraction, the reviewers will first discuss the information to be extracted to ensure clarity of the data extraction process. Any discrepancies between the reviewers will be resolved by discussion, and a third reviewer will be consulted if necessary. A custom-designed form will be developed in Excel for data charting. Two independent reviewers will pilot the data

For peer review only

charting form using a random sample of five included studies for consistency and required amendments will be agreed by consensus. We will modify the data extraction form as required based on feedback from the two reviewers, and the form amended at each stage where necessary. We plan to contact study authors in the case of unclear information and will make up to three attempts by email.

Data items

The following general information will be extracted and tabulated from the included articles: author name(s), publishing journal, year of publication, region in South Africa, study population, the study aims/objectives/question, the setting, study-design, and findings. In addition, the following data will be extracted according to the components of HSDF and the WHO's IPCH framework components. To ensure consistency of the data extraction process, we will consider the seven components using the descriptions in Table 1.

Data synthesis and analysis

The analytical framework will guide the data synthesis and thematic analysis. We anticipate that the dataset will include different study designs, and therefore both descriptive statistics and narrative synthesis will be used. We will establish the strengths and opportunities that should be optimised in order to improve UHC of stroke services in South Africa as well as the weaknesses and threats which need to be minimised or eliminated.

Availability of data and materials: All data generated or analysed during this study will be included in the published scoping review article.

Patient and Public Involvement:

No patients and public were not involved for the purpose of this scoping review.

Ethics and dissemination

Ethical approval is not required for this scoping review, as it will only include published and publicly available data. The findings of this review will be published in an open-access, peer-reviewed journal and we will develop an accessible summary of the results for website posting and stakeholder meetings. Data generated from this review will be made available upon reasonable request.

For peer review only

References

1. Scheffler E, Mash R. Surviving a stroke in South Africa: outcomes of home-based care in a low-resource rural setting. Topics in stroke rehabilitation. 2019 Aug 18;26(6):423-34.

2. Pillay-van Wyk V, Msemburi W, Laubscher R, Dorrington RE, Groenewald P, Glass T, Nojilana B, Joubert JD, Matzopoulos R, Prinsloo M, Nannan N. Mortality trends and differentials in South Africa from 1997 to 2012: second National Burden of Disease Study. Lancet Glob Health. 2016 Sep 1;4(9):e642-53.

3. Maredza M, Bertram MY, Gómez-Olivé XF, Tollman SM. Burden of stroke attributable to selected lifestyle risk factors in rural South Africa. BMC Public Health. 2016 Dec 1;16(1):143.

4. Coovadia H, Jewkes R, Barron P, Sanders D, McIntyre D. The health and health system of South Africa: historical roots of current public health challenges. The Lancet. 2009 Sep 5;374(9692):817-34.

5. Bertram MY, Katzenellenbogen J, Vos T, Bradshaw D, Hofman KJ. The disability adjusted life years due to stroke in South Africa in 2008. Int J Stroke. 2013 Oct;8(SA100):76-80.

6. Maredza M, Bertram MY, Tollman SM. Disease burden of stroke in rural South Africa: an estimate of incidence, mortality and disability adjusted life years. BMC Neurol 2015 Dec;15(1):54.

7. Salt is killing South Africans. The Heart and Stroke Foundation. 2015. https://www.health24.com/Diet-and-nutrition/News/Salt-is-killing-South-Africans-20130312.

8. Kabudula CW, Houle B, Collinson MA, Kahn K, Gómez-Olivé FX, Clark SJ, Tollman S. Progression of the epidemiological transition in a rural South African setting: findings from population surveillance in Agincourt, 1993–2013. BMC Public Health. 2017 Dec;17(1):424.

For peer review only

9. Yusuf S, Reddy S, Ôunpuu S, Anand S. Global burden of cardiovascular diseases: part I: general considerations, the epidemiologic transition, risk factors, and impact of urbanization. Circulation. 2001 Nov 27;104(22):2746-53.

10. Owolabi MO, Arulogun O, Melikam S, Adeoye AM, Akarolo-Anthony S, Akinyemi R, Arnett D, Tiwari H, Gebregziabher M, Jenkins C, Lackland D. The burden of stroke in Africa: a glance at the present and a glimpse into the future. Cardiovasc J Afr. 2015 Mar;26(2 H3Africa Suppl):S27.

11. Puchner KP, Rodriguez-Fernandez R, Oliver M, Solomos Z. Non-communicable diseases and tuberculosis: Anticipating the impending global storm. Glob Public Health. 2019 Sep 2;14(9):1372-81.

12. Zimba S, Ntanda PM, Lakhi S, Atadzhanov M. HIV infection, hypercoagulability and ischaemic stroke in adults at the University Teaching Hospital in Zambia: a case control study. BMC Infect Dis. 2017 Dec 1;17(1):354.

13. Heikinheimo T, Chimbayo D, Kumwenda JJ, Kampondeni S, Allain TJ. Stroke outcomes in Malawi, a country with high prevalence of HIV: a prospective follow-up study. PloS one. 2012;7(3).

14. Wu Z, McGoogan JM. Characteristics of and important lessons from the coronavirus disease 2019 (COVID-19) outbreak in China: summary of a report of 72 314 cases from the Chinese Center for Disease Control and Prevention. Jama. 2020 Apr 7;323(13):1239-42.

15. Kitzman P, Hudson K, Sylvia V, Feltner F, Lovins J. Care coordination for community transitions for individuals post-stroke returning to low-resource rural communities. Journal of community health. 2017 Jun 1;42(3):565-72.

16. Langhorne P, Bernhardt J, Kwakkel G. Stroke rehabilitation. The Lancet. 2011 May 14;377(9778):1693-702.

For peer review only

17. Lindsay MP, Norrving B, Furie KL, Donnan G, Langhorne P, Davis S. Global stroke guidelines and action plan: a road map for quality stroke care. Geneva: World Stroke Organization. 2016.
18. Miller KK, Lin SH, Neville M. From hospital to home to participation: a position paper on transition planning poststroke. Arch Phys Med Rehabil. 2019 Jun 1;100(6):1162-75.

19. Ntsiea MV. Current stroke rehabilitation services and physiotherapy research in South Africa. S Afr J Physiother. 2019;75(1).

20. Bryer A, Connor MD, Haug P, Cheyip B, Staub H, Tipping B, Duim WB, Pinkney-Atkinson V. The South African guideline for the management of ischemic stroke and transient ischemic attack: recommendations for a resource-constrained health care setting. Int J Stroke. 2011 Aug;6(4):349-54.

21. Rhoda A. *The rehabilitation of stroke patients at community health centres in the Western Cape* (Doctoral dissertation, University of the Western Cape).

22. South African Contextualised Stroke Rehabilitation Guideline (SA-CSRG). 2019. http://www.sun.ac.za/english/faculty/healthsciences/health-rehabilitation-sciences/Documents/Completed%20stroke%20guidelines_May2019.pdf

23. Mabunda SA, London L, Pienaar D. An evaluation of the role of an intermediate care facility in the continuum of care in Western Cape, South Africa. Int J Health Policy Manag. 2018 Feb;7(2):167.

24. Pandian JD, William AG, Kate MP, Norrving B, Mensah GA, Davis S, Roth GA, Thrift AG, Kengne AP, Kissela BM, Yu C. Strategies to improve stroke care services in low-and middle-income countries: a systematic review. Neuroepidemiology. 2017;49(1-2):45-61.

25. Visagie S, Swartz L. Rural South Africans' rehabilitation experiences: case studies from the Northern Cape Province. S Afr J Physiother. 2016;72(1).

26. Western Cape Government. Healthcare 2030: The Road to Wellness. Cape Town: Western Cape Department of Health; 2014.

For peer review only

27. Osokpo O, Riegel B. Cultural factors influencing self-care by persons with cardiovascular disease: An integrative review. Int J Nurs Stud. 2019 Jul 9:103383.

28. Benatar S, Gill S. Universal access to healthcare: the case of South Africa in the comparative global context of the late anthropocene era. International Journal of Health Policy and Management. 2020 Mar 1.

29. van den Heever AM. South Africa's universal health coverage reforms in the post-apartheid period. Health Policy. 2016 Dec 1;120(12):1420-8.

30. World Health Organisation: Tracking universal health coverage: 2017 global monitoring report. World Health Organization and International Bank for Reconstruction and Development / The World Bank; 2017. Licence: CC BY-NC-SA 3.0 IGO.

31. National Health Insurance Bill (https://www.gov.za/documents/national-health-insurance-bill-b-11-2019-6-aug-2019-0000)

32. McIntyre D, Garshong B, Mtei G, Meheus F, Thiede M, Akazili J, Ally M, Aikins M, Mulligan JA, Goudge J. Beyond fragmentation and towards universal coverage: insights from Ghana, South Africa and the United Republic of Tanzania. Bulletin of the World health Organization. 2008;86:871-6.

33. World Health Organisation: Health Systems Financing: The Path to Universal Coverage. World Health Report 2010. Geneva: WHO; 2010a.

34. Abiiro GA, De Allegri M. Universal health coverage from multiple perspectives: a synthesis of conceptual literature and global debates. BMC Int Health Hum Rights. 2015 Dec 1;15(1):17.

35. Fusheini A, Eyles J. Achieving universal health coverage in South Africa through a district health system approach: conflicting ideologies of health care provision. BMC Health Serv Res. 2016 Dec 1;16(1):558.

For peer review only

36. Louw Q, Twizeyemariya A, Grimmer K, Leibbrandt D. Estimating the costs and benefits of stroke rehabilitation in South Africa. J Eval Clin Pract. 2019 Sep 10.

37. Baatiema L, Otim M, Mnatzaganian G, Aikins AD, Coombes J, Somerset S. Towards best practice in acute stroke care in Ghana: a survey of hospital services. BMC Health Serv Res. 2017 Dec;17(1):108.

38. Cockburn L, Fanfon TN, Bramall A, Ngole EM, Kuwoh P, Anjonga E, Difang BM, Kiani S, Muso PS, Trivedi N, Sama J. Best practice guidelines for stroke in Cameroon: An innovative and participatory knowledge translation project. Afr J Disabil. 2014;3(1).

39. Okwen PM, Maweu I, Grimmer K, Margarita Dizon J. Evaluation of all African clinical practice guidelines for hypertension: Quality and opportunities for improvement. J Eval Clin Pract. 2019 Aug;25(4):565-74.

40. Grimmer K, Louw Q, Dizon JM, van Niekerk SM, Ernstzen D, Wiysonge C. Standardising evidence strength grading for recommendations from multiple clinical practice guidelines: a South African case study. Implementation Science. 2018 Dec 1;13(1):117.

41. Van Olmen J, Criel B, Bhojani U, Marchal B, Van Belle S, Chenge F, Hoeree T, Pirard M, Van Damme W, Kegels G. The health system dynamics framework: the introduction of an analytical model for health system analysis and its application to two case-studies. Health Culture and Society. 2012;2(1):1-21.

42. World Health Organization. Framework on integrated, people-centred health services. Report by the Secretariat. 69th World Health Assembly provisional agenda item. 2016 Apr 15;16:A69.

43. Levac D, Colquhoun H, O'Brien KK. Scoping studies: advancing the methodology. Implementation science. 2010 Dec 1;5(1):69.

44. Tricco AC, Lillie E, Zarin W, O'Brien KK, Colquhoun H, Levac D, Moher D, Peters MD, Horsley T, Weeks L, Hempel S. PRISMA extension for scoping reviews (PRISMA-ScR): checklist and explanation. Annals of internal medicine. 2018 Oct 2;169(7):467-73.

For peer review only

45. Mayosi BM, Lawn JE, Van Niekerk A, Bradshaw D, Karim SS, Coovadia HM, Lancet South Africa team. Health in South Africa: changes and challenges since 2009. The Lancet. 2012 Dec 8;380(9858):2029-43.

Contributions

SvN and GIJ drafted and revised the protocol with suggestions from TS, JW, SK, SF, RE and QAL who reviewed the protocol and provided feedback on the draft. SvN, SF and GIJ in consultation with the other authors constructed the search. All authors read and approved the final protocol.

Funding

This research was commissioned by the National Institute for Health Research (NIHR) NIHR Global Health Policy and Systems Research Development Award using UK aid from the UK Government. The views expressed in this publication are those of the author(s) and not necessarily those of the NIHR or the Department of Health and Social Care. Grant number NIHR130180.

Competing interests

None declared.

Figure Legends

Figure 1. Analytical framework (adapted from The Health Systems Dynamics Framework [41] and the WHO Framework on integrated people-centred health services components [42]

Table legends

Table 1: Application of the key characteristics of the analytical framework

Table 1: Application of the key characteristics of the analytical framework

FRAMEWORK COMPONENT	DESCRIPTION (data items)
**Governance/Regulation**	Healthcare policies at national or provincial levels; resource allocation policies; accountability; coordination and regulations.
**Resources**	Infrastructure (accessible; equipped and maintained); finance allocation and affordability; human resources (availability; distribution – occupation/specialisation, place of work, gender;

For peer review only

	graduates of health professionals); knowledge (clinical decision-making; clinical guidelines).
**Service Delivery**	Service delivery models; health services and service providers (private/public; for-profit or not–for-profit, formal or informal, professional or non-professional, allopathic or traditional, remunerated or voluntary).
**Context**	Socio-economic, technological, cultural, political and environmental environments.
**Re-orientation of care**	Health promotion and patient education. New technologies (eHealth; shared electronic medical records; telemedicine; m-health). Coordination of health services; continuous health care, multi-professional health teams; comprehensive health services and intersectoral coordination.
**Enabling environment**	Health workforce allocation/ shortages; distribution of health workforce; workforce training; clearly defined roles and fair wages.
**Population: Community engagement**	Engaging and empowering individuals, families, communities and informal carers. Reaching underserved and marginalised communities.<br>Common decision-making, self-efficacy of patients.

List of Abbreviations

UHC – Universal Health Coverage

SA – South Africa

WHO – World Health Organisation

IPCHS - Integrated people-centred health services

For peer review only

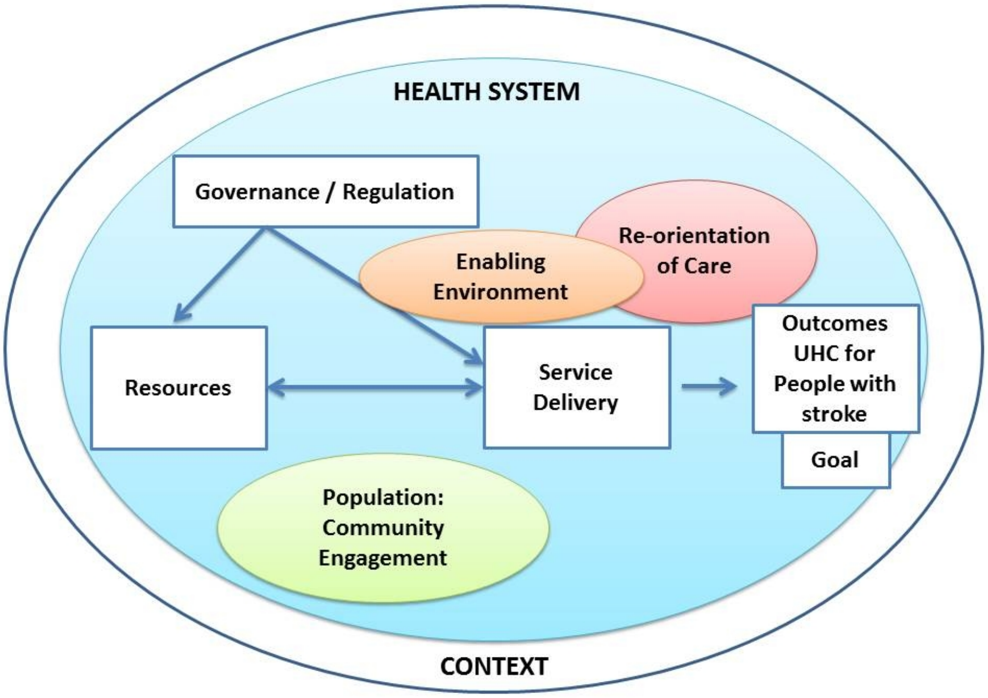

Figure 1. Analytical framework (adapted from The Health Systems Dynamics Framework [41] and the WHO Framework on integrated people-centered health services components [42]

254x190mm (96 x 96 DPI)

Achieving Universal Health Coverage for people with stroke in South Africa: Protocol for a scoping review

Supplementary File:

Definitions

Universal health coverage **-** "Universal health coverage means that all people receive the health services they need, including public health services designed to promote better health (such as anti-tobacco information campaigns and taxes), prevent illness (such as vaccinations), and to provide treatment, rehabilitation and palliative care (such as end-of-life care) of sufficient quality to be effective, while at the same time ensuring that the use of these services does not expose the user to financial hardship"[1].

Health System - "A health system consists of all organizations, people and actions whose *primary intent* is to promote, restore or maintain health. This includes efforts to influence the determinants of health as well as more direct health-improving activities. A health system is, therefore, more than the pyramid of publicly owned facilities that deliver personal health services"[2].

Health system goals – "Health systems have multiple goals. The World health report 2000 defined overall health system outcomes or goals such as improving health and health equity, in ways that are responsive, financially fair, and make the best, or most efficient, use of available resources. There are also important intermediate goals: the route from inputs to health outcomes is through achieving greater access to and coverage for effective health interventions, without compromising efforts to ensure provider quality and safety"[2].

Health System building blocks – "To achieve their goals, all health systems have to carry out some basic functions, regardless of how they are organized: they have to provide services; develop health workers and other key resources; mobilize and allocate finances, and ensure health system leadership and governance (also known as stewardship, which is about oversight and guidance of the whole system). For the purpose of clearly articulating what WHO will do to help strengthen health systems, the functions identified in the World health report 2000 have been broken down into a set of six essential 'building blocks'. All are needed to improve outcomes [2].

For peer review only

Health system strengthening - Is defined as improving the six health system building blocks and managing their interactions in ways that achieve more equitable and sustained improvements across health services and health outcomes. It requires both technical and political knowledge and action[2].

Stroke care- Is defined by the World Stroke Organisation as **"...**the continuum of care starting at the onset of a stroke event through the hyperacute phase, acute inpatient care, stroke rehabilitation, prevention of recurrent stroke and concludes with community reintegration and long term recovery" [3].

Clinical guideline - Clinical guidelines are statements that include recommendations intended to optimise patient care that are informed by a systematic review of evidence and an assessment of the benefits and harms of alternative care options [4].

References:

1. World Health Organisation: Tracking universal health coverage: 2017 global monitoring report. World Health Organization and International Bank for Reconstruction and Development / The World Bank; 2017. Licence: CC BY-NC-SA 3.0 IGO.

2. World Health Organization: Everybody's business: strengthening health systems to improve health outcomes. WHO's Framework for Action. Geneva: World Health Organization; 2007. http://wwwwhoint/healthsystems/strategy/everybodys_businesspdf.

3. Lindsay MP, Norrving B, Furie KL, Donnan G, Langhorne P, Davis S. Global stroke guidelines and action plan: a road map for quality stroke care. Geneva: World Stroke Organization. 2016.

4. Institute of Medicine (US). Committee on standards for developing trustworthy clinical practice guidelines. Clinical practice guidelines we can trust. 2011:53-6.

For peer review only

Achieving Universal Health Coverage for people with stroke in South Africa: Protocol for a scoping review

Supplementary File - 2

OVID Search

Database: Embase <1980 to 2020 Week 24>, Global Health <1910 to 2020 Week 23>, Journals@Ovid Full Text <June 17, 2020>, APA PsycExtra <1908 to June 08, 2020>, APA PsycInfo <1806 to June Week 2 2020>, LSHTM Journals@Ovid, Econlit <1886 to June 11, 2020>, Ovid MEDLINE(R) and Epub Ahead of Print, In-Process & Other Non-Indexed Citations, Daily and Versions(R) <1946 to June 16, 2020>, Social Policy and Practice <202004>
Search Strategy:

----

1 Stroke.mp. [mp=ti, ab, hw, tn, ot, dm, mf, dv, kw, fx, dq, bt, id, cc, tx, sh, ct, tc, tm, mh, nm, kf, ox, px, rx, an, ui, sy, pt] (1263546)
2 cerebro vascular accident.mp. [mp=ti, ab, hw, tn, ot, dm, mf, dv, kw, fx, dq, bt, id, cc, tx, sh, ct, tc, tm, mh, nm, kf, ox, px, rx, an, ui, sy, pt] (560)
3 ischaemia.mp. [mp=ti, ab, hw, tn, ot, dm, mf, dv, kw, fx, dq, bt, id, cc, tx, sh, ct, tc, tm, mh, nm, kf, ox, px, rx, an, ui, sy, pt] (140097)
4 hemorrhage.mp. [mp=ti, ab, hw, tn, ot, dm, mf, dv, kw, fx, dq, bt, id, cc, tx, sh, ct, tc, tm, mh, nm, kf, ox, px, rx, an, ui, sy, pt] (923222)
5 Universal health coverage.mp. [mp=ti, ab, hw, tn, ot, dm, mf, dv, kw, fx, dq, bt, id, cc, tx, sh, ct, tc, tm, mh, nm, kf, ox, px, rx, an, ui, sy, pt] (9467)
6 universal access.mp. [mp=ti, ab, hw, tn, ot, dm, mf, dv, kw, fx, dq, bt, id, cc, tx, sh, ct, tc, tm, mh, nm, kf, ox, px, rx, an, ui, sy, pt] (12850)
7 Universal health care.mp. [mp=ti, ab, hw, tn, ot, dm, mf, dv, kw, fx, dq, bt, id, cc, tx, sh, ct, tc, tm, mh, nm, kf, ox, px, rx, an, ui, sy, pt] (7370)
8 universal health access.mp. [mp=ti, ab, hw, tn, ot, dm, mf, dv, kw, fx, dq, bt, id, cc, tx, sh, ct, tc, tm, mh, nm, kf, ox, px, rx, an, ui, sy, pt] (64)
9 stroke disability.mp. [mp=ti, ab, hw, tn, ot, dm, mf, dv, kw, fx, dq, bt, id, cc, tx, sh, ct, tc, tm, mh, nm, kf, ox, px, rx, an, ui, sy, pt] (1581)
10 stroke rehabilitation.mp. [mp=ti, ab, hw, tn, ot, dm, mf, dv, kw, fx, dq, bt, id, cc, tx, sh, ct, tc, tm, mh, nm, kf, ox, px, rx, an, ui, sy, pt] (30385)
11 stroke care.mp. [mp=ti, ab, hw, tn, ot, dm, mf, dv, kw, fx, dq, bt, id, cc, tx, sh, ct, tc, tm, mh, nm, kf, ox, px, rx, an, ui, sy, pt] (18395)
12 treatment access.mp. [mp=ti, ab, hw, tn, ot, dm, mf, dv, kw, fx, dq, bt, id, cc, tx, sh, ct, tc, tm, mh, nm, kf, ox, px, rx, an, ui, sy, pt] (6626)
13 health systems.mp. [mp=ti, ab, hw, tn, ot, dm, mf, dv, kw, fx, dq, bt, id, cc, tx, sh, ct, tc, tm, mh, nm, kf, ox, px, rx, an, ui, sy, pt] (111350)
14 South Africa.mp. [mp=ti, ab, hw, tn, ot, dm, mf, dv, kw, fx, dq, bt, id, cc, tx, sh, ct, tc, tm, mh, nm, kf, ox, px, rx, an, ui, sy, pt] (235927)
15 Eastern Cape.mp. [mp=ti, ab, hw, tn, ot, dm, mf, dv, kw, fx, dq, bt, id, cc, tx, sh, ct, tc, tm, mh, nm, kf, ox, px, rx, an, ui, sy, pt] (4355)
16 Free State.mp. [mp=ti, ab, hw, tn, ot, dm, mf, dv, kw, fx, dq, bt, id, cc, tx, sh, ct, tc, tm, mh, nm, kf, ox, px, rx, an, ui, sy, pt] (14110)
17 Gauteng.mp. [mp=ti, ab, hw, tn, ot, dm, mf, dv, kw, fx, dq, bt, id, cc, tx, sh, ct, tc, tm, mh, nm, kf, ox, px, rx, an, ui, sy, pt] (4356)
18 KwaZulu-Natal.mp. [mp=ti, ab, hw, tn, ot, dm, mf, dv, kw, fx, dq, bt, id, cc, tx, sh, ct, tc, tm, mh, nm, kf, ox, px, rx, an, ui, sy, pt] (11901)

For peer review only

19 Limpopo.mp. [mp=ti, ab, hw, tn, ot, dm, mf, dv, kw, fx, dq, bt, id, cc, tx, sh, ct, tc, tm, mh, nm, kf, ox, px, rx, an, ui, sy, pt] (3368)
20 Mpumalanga.mp. [mp=ti, ab, hw, tn, ot, dm, mf, dv, kw, fx, dq, bt, id, cc, tx, sh, ct, tc, tm, mh, nm, kf, ox, px, rx, an, ui, sy, pt] (1992)
21 Northern Cape.mp. [mp=ti, ab, hw, tn, ot, dm, mf, dv, kw, fx, dq, bt, id, cc, tx, sh, ct, tc, tm, mh, nm, kf, ox, px, rx, an, ui, sy, pt] (997)
22 North West.mp. [mp=ti, ab, hw, tn, ot, dm, mf, dv, kw, fx, dq, bt, id, cc, tx, sh, ct, tc, tm, mh, nm, kf, ox, px, rx, an, ui, sy, pt] (39969)
23 Western Cape.mp. [mp=ti, ab, hw, tn, ot, dm, mf, dv, kw, fx, dq, bt, id, cc, tx, sh, ct, tc, tm, mh, nm, kf, ox, px, rx, an, ui, sy, pt] (8316)
24 1 or 2 or 3 or 4 (2162628)
25 5 or 6 or 7 or 8 (28371)
26 9 or 10 or 11 or 12 (54898)
27 15 or 16 or 17 or 18 or 19 or 20 or 21 or 22 or 23 (83478)
28 14 or 27 (286527)
29 13 or 25 (135636)
30 24 and 29 (7926)
31 24 and 26 and 29 (655)
32 24 and 28 and 29 (433)

****

For peer review only

UHC for Stroke care in SA - Scopus Search results

(Stroke OR Universal health coverage) AND (Health system) AND (LIMIT-TO (AFFILCOUNTRY,"South Africa")) AND (LIMIT-TO (PUBYEAR,2020) OR LIMIT-TO (PUBYEAR,2019) OR LIMIT-TO (PUBYEAR,2018) OR LIMIT-TO (PUBYEAR,2017) OR LIMIT-TO (PUBYEAR,2016) OR LIMIT-TO (PUBYEAR,2015) OR LIMIT-TO (PUBYEAR,2014) OR LIMIT-TO (PUBYEAR,2013) OR LIMIT-TO (PUBYEAR,2012) OR LIMIT-TO (PUBYEAR,2011) OR LIMIT-TO (PUBYEAR,2010) OR LIMIT-TO (PUBYEAR,2009) OR LIMIT-TO (PUBYEAR,2008) OR LIMIT-TO (PUBYEAR,2007) OR LIMIT-TO (PUBYEAR,2006) OR LIMIT-TO (PUBYEAR,2004) OR LIMIT-TO (PUBYEAR,2003) OR LIMIT-TO (PUBYEAR,2002) OR LIMIT-TO (PUBYEAR,2000)) AND (LIMIT-TO (openaccess,1)) AND (LIMIT-TO (SUBJAREA,"MEDI") OR LIMIT-TO (SUBJAREA,"SOCI") OR LIMIT-TO (SUBJAREA,"MULT") OR LIMIT-TO (SUBJAREA,"NURS") OR LIMIT-TO (SUBJAREA,"HEAL") OR LIMIT-TO (SUBJAREA,"ENVI") OR LIMIT-TO (SUBJAREA,"PSYC") OR LIMIT-TO (SUBJAREA,"ECON") OR LIMIT-TO (SUBJAREA,"ARTS") OR LIMIT-TO (SUBJAREA,"NEUR")) AND (LIMIT-TO (PUBSTAGE,"final") OR LIMIT-TO (PUBSTAGE,"aip"))

For peer review only

		Narrow by SubjectThesaurus: - qualitative research<br>Narrow by SubjectThesaurus: - primary care<br>Narrow by SubjectThesaurus: - evaluation research<br>Narrow by SubjectThesaurus: - health behavior<br>Narrow by SubjectThesaurus: - community health services<br>Narrow by SubjectThesaurus: - health outcome assessment<br>Narrow by SubjectThesaurus: - government policy<br>Narrow by SubjectThesaurus: - world health<br>Narrow by SubjectThesaurus: - quality of life<br>Narrow by SubjectThesaurus: - health services accessibility<br>Narrow by SubjectThesaurus: - health promotion<br>Narrow by SubjectThesaurus: - public health<br>Search modes - Boolean/Phrase		
S5	(stroke or cerebrovascular accident or cva or cerebral vascular event or cve or transient ischaemic attack or tia) OR (stroke rehabilitation or stroke recovery) OR stroke	Limiters - Published Date: 20050101-20200631<br>Expanders - Apply related words; Apply equivalent subjects<br>Narrow by Language: - english	Interface - EBSCOhost Research Databases<br>Search Screen - Advanced Search<br>Database - Academic Search Complete	16,739

For peer review only - http://bmjopen.bmj.com/site/about/guidelines.xhtml

prevention OR stroke patients AND universal health care OR universal health coverage OR universal health coverage in south africa AND health system OR health systems strengthening OR (health system or health services) OR health systems management AND south africa	Narrow by SubjectThesaurus: - public health surveillance Narrow by SubjectThesaurus: - primary health care Narrow by SubjectThesaurus: - national health services Narrow by SubjectThesaurus: - health insurance Narrow by SubjectThesaurus: - health disparities Narrow by SubjectThesaurus: - health care reform Narrow by SubjectThesaurus: - health programs Narrow by SubjectThesaurus: - chronic diseases Narrow by SubjectThesaurus: - economics Narrow by SubjectThesaurus: - systematic reviews (medical research) Narrow by SubjectThesaurus: - qualitative research Narrow by SubjectThesaurus: - primary care Narrow by SubjectThesaurus: - evaluation research Narrow by SubjectThesaurus: - health behavior Narrow by SubjectThesaurus: - community health services Narrow by SubjectThesaurus: -

		health outcome assessment<br>Narrow by SubjectThesaurus: - government policy<br>Narrow by SubjectThesaurus: - world health<br>Narrow by SubjectThesaurus: - quality of life<br>Narrow by SubjectThesaurus: - health services accessibility<br>Narrow by SubjectThesaurus: - health promotion<br>Narrow by SubjectThesaurus: - public health<br>Search modes - Boolean/Phrase		
S4	(stroke or cerebrovascular accident or cva or cerebral vascular event or cve or transient ischaemic attack or tia) OR (stroke rehabilitation or stroke recovery) OR stroke prevention OR stroke patients AND universal health care OR universal health coverage OR universal health coverage in south africa AND health system OR health systems strengthening OR (health system or health services) OR health systems management AND south africa	Limiters - Published Date: 20050101-20200631<br>Expanders - Apply related words; Apply equivalent subjects<br>Narrow by SubjectThesaurus: - public health surveillance<br>Narrow by SubjectThesaurus: - primary health care<br>Narrow by SubjectThesaurus: - national health services<br>Narrow by SubjectThesaurus: - health insurance<br>Narrow by SubjectThesaurus: - health disparities<br>Narrow by SubjectThesaurus: - health care reform<br>Narrow by SubjectThesaurus: -	Interface - EBSCOhost Research Databases<br>Search Screen - Advanced Search<br>Database - Academic Search Complete	17,251

health programs
Narrow by
SubjectThesaurus: -
chronic diseases
Narrow by
SubjectThesaurus: -
economics
Narrow by
SubjectThesaurus: -
systematic reviews
(medical research)
Narrow by
SubjectThesaurus: -
qualitative research
Narrow by
SubjectThesaurus: -
primary care
Narrow by
SubjectThesaurus: -
evaluation research
Narrow by
SubjectThesaurus: -
health behavior
Narrow by
SubjectThesaurus: -
community health
services
Narrow by
SubjectThesaurus: -
health outcome
assessment
Narrow by
SubjectThesaurus: -
government policy
Narrow by
SubjectThesaurus: -
world health
Narrow by
SubjectThesaurus: -
quality of life
Narrow by
SubjectThesaurus: -
health services
accessibility
Narrow by
SubjectThesaurus: -
health promotion
Narrow by
SubjectThesaurus: -

For peer review only - http://bmjopen.bmj.com/site/about/guidelines.xhtml

		public health<br>Search modes - Boolean/Phrase		
S3	(stroke or cerebrovascular accident or cva or cerebral vascular event or cve or transient ischaemic attack or tia) OR (stroke rehabilitation or stroke recovery) OR stroke prevention OR stroke patients AND universal health care OR universal health coverage OR universal health coverage in south africa AND health system OR health systems strengthening OR (health system or health services) OR health systems management AND south africa	Limiters - Published Date: 20050101-20200631<br>Expanders - Apply related words; Apply equivalent subjects<br>Narrow by SubjectThesaurus: - public health<br>Search modes - Boolean/Phrase	Interface - EBSCOhost Research Databases<br>Search Screen - Advanced Search<br>Database - Academic Search Complete	84,912
S2	(stroke or cerebrovascular accident or cva or cerebral vascular event or cve or transient ischaemic attack or tia) OR (stroke rehabilitation or stroke recovery) OR stroke prevention OR stroke patients AND universal health care OR universal health coverage OR universal health coverage in south africa AND health system OR health systems strengthening OR (health system or health services) OR health systems management AND south africa	Limiters - Published Date: 20050101-20200631<br>Expanders - Apply related words; Apply equivalent subjects<br>Search modes - Boolean/Phrase	Interface - EBSCOhost Research Databases<br>Search Screen - Advanced Search<br>Database - Academic Search Complete	878,271
S1	(stroke or cerebrovascular accident or cva or cerebral	Limiters - Published Date: 20050101-20200631	Interface - EBSCOhost Research Databases<br>Search Screen - Advanced	878,271

vascular event or cve or transient ischaemic attack or tia) OR (stroke rehabilitation or stroke recovery) OR stroke prevention OR stroke patients AND universal health care OR universal health coverage OR universal health coverage in south africa AND health system OR health systems strengthening OR (health system or health services) OR health systems management OR health system financing	Expanders - Apply related words; Apply equivalent subjects Search modes - Boolean/Phrase	Search Database - Academic Search Complete
